# A theoretical comparison between two ruminal electron sinks

**DOI:** 10.3389/fmicb.2013.00319

**Published:** 2013-10-30

**Authors:** Emilio M. Ungerfeld

**Affiliations:** CONICYT Regional R10C1002, Centro de Investigación y Desarrollo CIEN Austral, Universidad Austral de ChilePuerto Montt, Chile

**Keywords:** rumen, methane, hydrogen, fermentation, reductive acetogenesis, propionate, ruminant nutrition

## Abstract

Dihydrogen accumulation resulting from methanogenesis inhibition in the rumen is an energy loss and can inhibit fermentation. The objective of this analysis was to compare the energetic and nutritional consequences of incorporating H_2_ into reductive acetogenesis or additional propionate production beyond the acetate to propionate shift occurring along with methanogenesis inhibition. Stoichiometric consequences were calculated for a simulated fermentation example. Possible nutritional consequences are discussed. Incorporating H_2_ into reductive acetogenesis or additional propionate production resulted in equal heat of combustion output in volatile fatty acids (VFA). Incorporation of H_2_ into reductive acetogenesis could result in moderate decrease in ruminal pH, although whole-animal buffering mechanisms make pH response difficult to predict. Research would be needed to compare the microbial protein production output. There could be post-absorptive implications due to differences in VFA profile. Electron incorporation into reductive acetogenesis could favor energy partition toward milk, but increase risk of ketosis in high-producing dairy cows on ketogenic diets. Greater propionate production could favor milk protein production, but may be less desirable in animals whose intake is metabolically constrained, like feedlot steers. Because of the different nutritional implications, and because practical solutions to incorporate H_2_ into either pathway are not yet available, it is recommended to research both alternatives.

## Introduction

**Figure 1:**
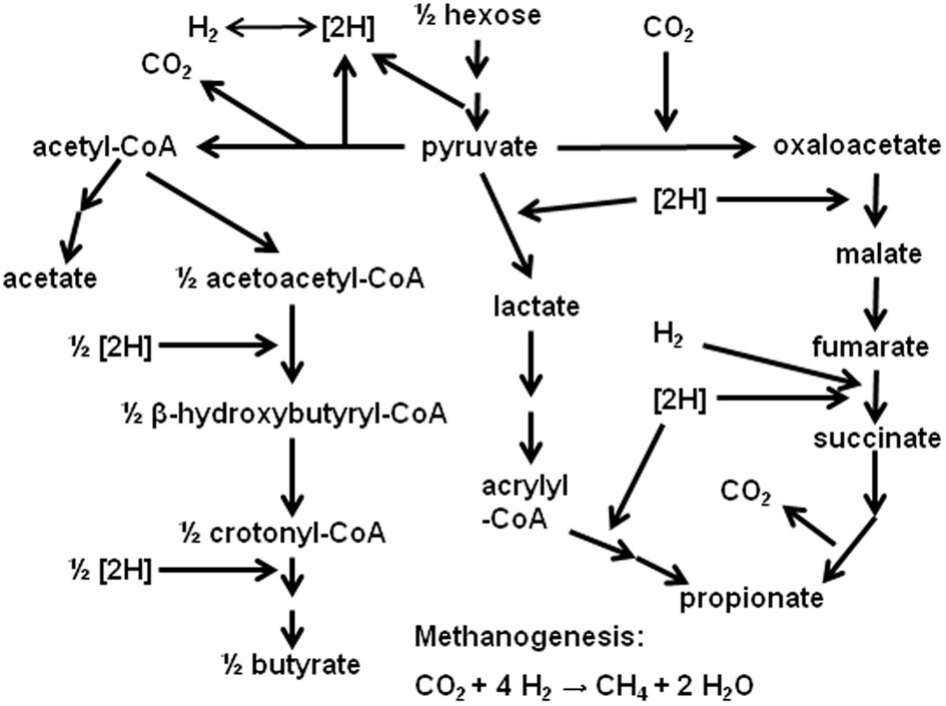
**Main ruminal fermentation pathways**.

In ruminal fermentation, H_2_ is produced from reducing equivalents released in glycolysis and pyruvate oxidative decarboxylation to acetyl-CoA (Figure 1). Methane is the main sink for H_2_ electrons in the rumen (Wolin et al., 1997). Interspecies H_2_ transfer from the fermentative community of bacteria, protozoa and fungi to methanogens is of great significance. Methanogenesis allows maintaining a low H_2_ pressure, shifting fermentation away from ethanol and lactate and toward acetate, which allows extra ATP generation by substrate level phosphorylation in acetate formation, and by electron transport-linked-phosphorylation in methanogenesis itself (Russell and Wallace, 1997; Wolin et al., 1997). However, despite its profound implication to ruminal fermentation, CH_4_ formation constitutes a loss of between 2 and 12% of the gross energy consumed by ruminants (Attwood and McSweeney, 2008). Also, CH_4_ emission by livestock is an important source of anthropogenic greenhouse gases emissions (Morgavi et al., 2010).

Because of these environmental and animal production efficiency issues, considerable research on the inhibition of ruminal methanogenesis has been conducted. Inhibition of methanogenesis poses the problem of the incorporation of the electrons not used in CH_4_ formation into alternative pathways. As methanogenesis is inhibited, H_2_ pressure increases, and this thermodynamically favors electron incorporation into propionate (Figure 1; Janssen, 2010). Nevertheless, incorporation of reducing equivalents spared from methanogenesis into propionate is not complete (Czerkawski, 1986), and H_2_ accumulates, especially if CH_4_ production is strongly inhibited, whether *in vitro* (e.g., Trei et al., 1971; Stanier and Davies, 1981; Sauer and Teather, 1987) or *in vivo* (e.g., Trei et al., 1971; Nollet et al., 1998; Kung et al., 2003; Mitsumori et al., 2012).

It is therefore necessary to conduct research on the incorporation of accumulated H_2_ into electron sinks nutritionally useful to the animal. In order to better guide current research efforts in this area, it would be important to anticipate the outcomes of incorporating H_2_ into different electron sinks. The present theoretical analysis compares the hypothetical energetic and nutritional consequences of incorporating accumulated H_2_ into reductive acetogenesis or additional propionate formation beyond the typical acetate to propionate shift that occurs when methanogenesis is inhibited. Dihydrogen incorporation into a useful electron sink is a necessary part of an integral methanogenesis-inhibition strategy, because it minimizes gaseous digestible energy (DE) losses and avoids fermentation inhibition. Thus, it is important to predict, compare and anticipate how, not only fermentation, but whole animal digestion and metabolism, and hence production, could change if H_2_ was incorporated into different alternative electron sinks.

Electron-incorporating processes alternative to methanogenesis other than reductive acetogenesis and propionate production will not be considered in the present analysis. Nitrate and sulphate reduction (Van Zijderveld et al., 2010), and reduction of oxygen entering the rumen through the rumen wall and in feed and water (Czerkawski, 1986) do not incorporate electrons into energy sources available to ruminants (although nitrate could replace urea as a source of N on an equal moles of N basis). Long chain fatty acids biohydrogenation only uses a small proportion of metabolic hydrogen produced in fermentation (Czerkawski, 1986). Microbial biomass synthesis also requires reducing power (Hungate et al., 1961), and both theoretical considerations (Czerkawski, 1986) and *in vitro* results (Ungerfeld et al., 2007; Guo et al., 2009) suggests that it can be an important alternative electron sink to CH_4_; however, consequences of methanogenesis inhibition on microbial growth are still incompletely quantified and understood as they involve multiple anabolic pathways in a complex microbial community, and this electron sink is therefore not compared to reductive acetogenesis and propionate production in this analysis.

## Procedures

### Stoichiometric calculations

#### Typical ruminal fermentation

Both starch and cellulose are hydrolyzed to glucose; however, the fermentation pattern of starch is lower in acetate to propionate ratio compared to cellulose (Bannink et al., 2006). Because acetate production results in the release of reducing equivalents, whereas propionate production incorporates them (Figure 1), fermentation of concentrates typically associates with less CH_4_ produced per mol of hexose fermented, as propionate production competes with methanogenesis for reducing equivalents (Czerkawski, 1986; Wolin et al., 1997). Two typical example fermentation stoichiometries for roughage- (Equation 1) and concentrate-based (Equation 2) diets are provided below. In Equation 1 (roughage), 1 mol of glucose is fermented to VFA and gases with 4 to 1 acetate to propionate ratio; in Equation 2 (concentrate), 1 mol of glucose is fermented to VFA and gases with 1.5 to 1 acetate to propionate ratio:

(1) $$\begin{array}{l} {\text{C}}_{6}{\text{H}}_{12}{\text{O}}_{6}\to 4/3{\text{CH}}_{3}{\text{COO}}^{-}+1/3{\text{CH}}_{3}{\text{CH}}_{2}{\text{COO}}^{-}\\ \;+1/6{\text{CH}}_{3}{\text{CH}}_{2}{\text{CH}}_{2}{\text{COO}}^{-}+11/6{\text{H}}^{+}\\ \;+{\text{CO}}_{2}+2/3{\text{CH}}_{4}+1/3{\text{H}}_{2}\text{O} \end{array}$$

(2) $$\begin{array}{l} {\text{C}}_{6}{\text{H}}_{12}{\text{O}}_{6}\to 12/13{\text{CH}}_{3}{\text{COO}}^{-}+8/13{\text{CH}}_{3}{\text{CH}}_{2}{\text{COO}}^{-}\\ \;+3/13{\text{CH}}_{3}{\text{CH}}_{2}{\text{CH}}_{2}{\text{COO}}^{-}+23/13{\text{H}}^{+}\\ \;+25/26{\text{CO}}_{2}+11/26{\text{CH}}_{4}+7/13{\text{H}}_{2}\text{O} \end{array}$$

**Figure 2:**
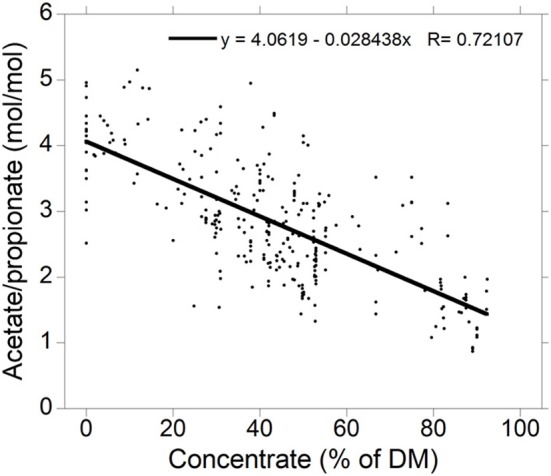
**Relationship between the acetate to propionate molar concentration ratio and the percentage of concentrate in the diet dry matter (DM) for 300 treatment means in 73 experiments (Ungerfeld, unpublished)**.

Acetate to propionate ratios for high-roughage and high-concentrate diets were obtained from a compilation of 300 treatment means from 79 *in vivo* studies in which ruminal VFA concentrations were reported (Figure 2; Ungerfeld, unpublished). Production of valerate, caproate and branched-chain VFA are not considered for the sake of simplification. It is also important to note that VFA proportions are also affected by factors other than the chemical fraction fermented (Bannink et al., 2008).

#### Methanogenesis-inhibited ruminal fermentation

When methanogenesis is inhibited, reducing equivalents are diverted into alternative electron sinks. Some of these alternative electron sinks are typical to ruminal fermentation, like propionate (Czerkawski, 1986; Wolin et al., 1997; Janssen, 2010) and microbial growth (Czerkawski, 1986; Ungerfeld et al., 2007; Guo et al., 2009); in contrast, intermediate metabolites that are usually present at very low concentration or pressure, like H_2_ (Janssen, 2010) or format (Czerkawski, 1986), accumulate. A hypothetical situation in which methanogenesis in the roughage diet of Equation 1 is inhibited by 92% (similar extent of inhibition: Nollet et al., 1997a; Goel et al., 2009; Tomkins et al., 2009; Mitsumori et al., 2012), with resulting H_2_ accumulation, is represented in Equation 3:

(3) $$\begin{array}{l} {\text{C}}_{6}{\text{H}}_{12}{\text{O}}_{6}+3/5\;{\text{H}}_{2}\text{O}+66\text{X}/59\;\to 70/59\;{\text{CH}}_{3}{\text{COO}}^{-}\\ \;+28/59\;{\text{CH}}_{3}{\text{CH}}_{2}{\text{COO}}^{-}+10/59\;{\text{CH}}_{3}{\text{CH}}_{2}{\text{CH}}_{2}{\text{COO}}^{-}\\ \;+108/59\;{\text{H}}^{+}+867/590{\text{CO}}_{2}+264/295\;{\text{H}}_{2}+33/590\;{\text{CH}}_{4}\\ \;+66\text{X}[2\text{H}]/59 \end{array}$$

The sum of electron sinks other than CH_4_, propionate and H_2_ (i.e., mainly microbial biomass and formate) are represented in Equation 3 by the term X[2H], accounting in this example for 32% of total electron-pairs incorporated. The shift in the acetate to propionate ratio from 4 to 1 to 2.5 to 1 in Equation 3 with respect to Equation 1 resulted in propionate formation increasing its participation as an electron sink from 18% in Equation 1 to 27% in Equation 3; similar shifts in the acetate to propionate ratio as a consequence of methanogenesis inhibition were reported by Ungerfeld et al. (2003, 2006) *in vitro* and Mitsumori et al. (2012) *in vivo*. Participation of CH_4_ as electron sink decreased from 73 to 6%, and the remaining 33% electron-pairs accumulated as H_2_ (calculations not shown). If the amount of hexose fermented does not change, C in the extra microbial biomass would come partly from less C in fermentation products (Dijkstra et al., 2007). The C shift from fermentation products to microbial biomass was not considered in Equation 3 in order to maintain the C balance explicit between fermented hexose and VFA and gases.

It is important to consider that, although the stoichiometric changes chosen are arbitrary, they do not affect the energetic and nutritional comparison that will be conducted between reductive acetogenesis and additional propionate as potential electron sinks for the accumulated H_2_. This is because, for any fermentation stoichiometry alternative to Equation 3 that could be chosen, the simulation that will be conducted would still compare the outcome of incorporating the same amount of accumulated H_2_ into either reductive acetogenesis or additional propionate formation.

#### Reductive acetogenesis

Reductive acetogenesis involves the reduction of CO_2_ by H_2_ to acetate:

(4) $$2\;{\text{CO}}_{2}+4\;{\text{H}}_{2}\to {\text{CH}}_{3}{\text{COO}}^{-}+{\text{H}}^{+}+2\;{\text{H}}_{2}\text{O}$$

Multiples of Equation 3 and 4 can be added up to result in different proportions of the accumulated H_2_ of Equation 3 being converted to acetate through reductive acetogenesis. Because H_2_ in Equation 3 is limiting in its ratio to CO_2_ in relation to the H_2_ to CO_2_ stoichiometry in Equation 4, reductive acetogenesis could, in theory, incorporate 100% of the accumulated H_2_ in Equation 3:

(5) $$\begin{array}{l} {\text{C}}_{6}{\text{H}}_{12}{\text{O}}_{6}+9/59\;{\text{H}}_{2}\text{O}+66\text{X}/59\to 416/295\;{\text{CH}}_{3}{\text{COO}}^{-}\\ \;+28/59\;{\text{CH}}_{3}{\text{CH}}_{2}{\text{COO}}^{-}+10/59\;{\text{CH}}_{3}{\text{CH}}_{2}{\text{CH}}_{2}{\text{COO}}^{-}\\ \;+606/295\;{\text{H}}^{+}+603/590\;{\text{CO}}_{2}+33/590\;{\text{CH}}_{4}\\ \;+66\text{X}[2\text{H}]/59 \end{array}$$

Total incorporation of H_2_ is a theoretical limit; even in the typical ruminal fermentation with functional methanogenesis, H_2_ concentration varies between 0.1 and 50 μM (Janssen, 2010). Generalizing Equation 5, incorporation of a positive fraction of accumulated H_2_ smaller or equal than unity into reductive acetogenesis is equal to:

(6) $$\begin{array}{l} {\text{C}}_{6}{\text{H}}_{12}{\text{O}}_{6}\text{​}+\text{​}(177-132y)/295\;{\text{H}}_{2}\text{O}+66\text{X}/59\text{​}\to \\ \;(350+66y)/295\;{\text{CH}}_{3}{\text{COO}}^{-}+28/59\;{\text{CH}}_{3}{\text{CH}}_{2}{\text{COO}}^{-}\\ \;+10/59\;{\text{CH}}_{3}{\text{CH}}_{2}{\text{CH}}_{2}{\text{COO}}^{-}+(540+66y)/295\;{\text{H}}^{+}\\ \;+(867-264y)/590\;{\text{CO}}_{2}+264(1-y)/295\;{\text{H}}_{2}\\ \;+33/590\;{\text{CH}}_{4}+66\text{X}[2\text{H}]/59 \end{array}$$

where *y* is the proportion of accumulated H_2_ incorporated into reductive acetogenesis, 0 ≤ *y* ≤ 1.

#### Propionate formation

Some propionate producers utilize H_2_ for reducing fumarate to succinate (Figure 1; Henderson, 1980). In agreement, an increase in H_2_ concentration thermodynamically stimulates propionate production (Janssen, 2010). Thus, accumulated H_2_ could, theoretically, be also utilized toward the formation of more propionate beyond the acetate to propionate shift typically associated to the inhibition of ruminal methanogenesis (i.e., further than the acetate to propionate shift of Equation 3 with respect to Equation 1). If the amount of fermented hexoses remains constant, C utilized to produce additional propionate would represent C in fermented hexoses not metabolized to other VFA or not used in anabolism. In the present analysis, it will be assumed that each extra mol of propionate is formed at the expense of 1 mol of acetate that is not produced, i.e., an acetate to propionate fermentation shift beyond that one typically observed associated with methanogenesis inhibition. For each mol of acetate that is not produced, 2 mol of reducing equivalents-pairs are not released:

(7) $$\begin{array}{l} {\text{C}}_{6}{\text{H}}_{12}{\text{O}}_{6}+2\;{\text{H}}_{2}O\text{​}\to \text{​}2\;{\text{CH}}_{3}{\text{COO}}^{-}+2{\text{H}}^{+}\\ \;+2\;{\text{CO}}_{2}+4\times [2\text{H}] \end{array}$$

Then, propionate formation competes for C with its major source of reducing equivalents, acetate formation. The acetate to propionate fermentation shift that redirects C and H_2_ toward propionate production is depicted in Equation 8:

(8) $${\text{CH}}_{3}{\text{COO}}^{-}+{\text{CO}}_{2}+3\;{\text{H}}_{2}\to {\text{CH}}_{3}{\text{CH}}_{2}{\text{COO}}^{-}+2\;{\text{H}}_{2}\text{O}$$

Incorporation of 100% of accumulated H_2_ in Equation 3 into propionate formation would result in:

(9) $$\begin{array}{l} {\text{C}}_{6}{\text{H}}_{12}{\text{O}}_{6}+1/295\;{\text{H}}_{2}\text{O}+66\text{X}/59\to 262/295\;{\text{CH}}_{3}{\text{COO}}^{-}\\ \;+228/295\;{\text{CH}}_{3}{\text{CH}}_{2}{\text{COO}}^{-}+10/59\;{\text{CH}}_{3}{\text{CH}}_{2}{\text{CH}}_{2}{\text{COO}}^{-}\\ \;+108/59\;{\text{H}}^{+}+691/590\;{\text{CO}}_{2}+33/590\;{\text{CH}}_{4}\\ \;+66\text{X}[2\text{H}]/59 \end{array}$$

Equation 9 generalizes for any positive fraction of accumulated H_2_ smaller or equal than unity incorporated into extra propionate (*z*) as:

(10) $$\begin{array}{l} {\text{C}}_{6}{\text{H}}_{12}{\text{O}}_{6}+(177-176z)/295\;{\text{H}}_{2}\text{O}+66X/59\to \\ \;(350-88z)/295\;{\text{CH}}_{3}{\text{COO}}^{-}\text{​}+\text{​}(140+88z)/295\;{\text{CH}}_{3}{\text{CH}}_{2}{\text{COO}}^{-}\\ \;+10/59\;{\text{CH}}_{3}{\text{CH}}_{2}{\text{CH}}_{2}{\text{COO}}^{-}\text{​}+\text{​}108/59\;{\text{H}}^{+}+(867-176z)/590\;{\text{CO}}_{2}\\ \;+264(1-z)/295\;{\text{H}}_{2}\text{​}+\text{​}33/590\;{\text{CH}}_{4}+66\text{X}[2\text{H}]/59 \end{array}$$

where 0 ≤ *z* ≤ 1.

### Simulated fermentation

Stoichiometric responses in production of VFA and gases per mole of hexose fermented to H_2_ incorporation into reductive acetogenesis or additional propionate, were calculated as *y* or *z* in Equation 6 and 10, respectively, varied between 0 and 1. The kinetics of reductive acetogenesis or additional propionate production, if they were thermodynamically feasible, could vary under different conditions. This analysis does not specify an incubation timeframe (if a batch culture system) or a fermentation rate (if a continuous culture system). Also, it was assumed that incorporation of electrons into other processes remained constant as accumulated H_2_ was incorporated into reductive acetogenesis or propionate formation. Results with total incorporation of accumulated H_2_ into reductive acetogenesis or additional propionate production (i.e., Equation 5 and 9, respectively) were also compared to the corresponding roughage fermentation with CH_4_ as main electron sink and no H_2_ accumulation (Equation 1).

## Results

**Figure 3:**
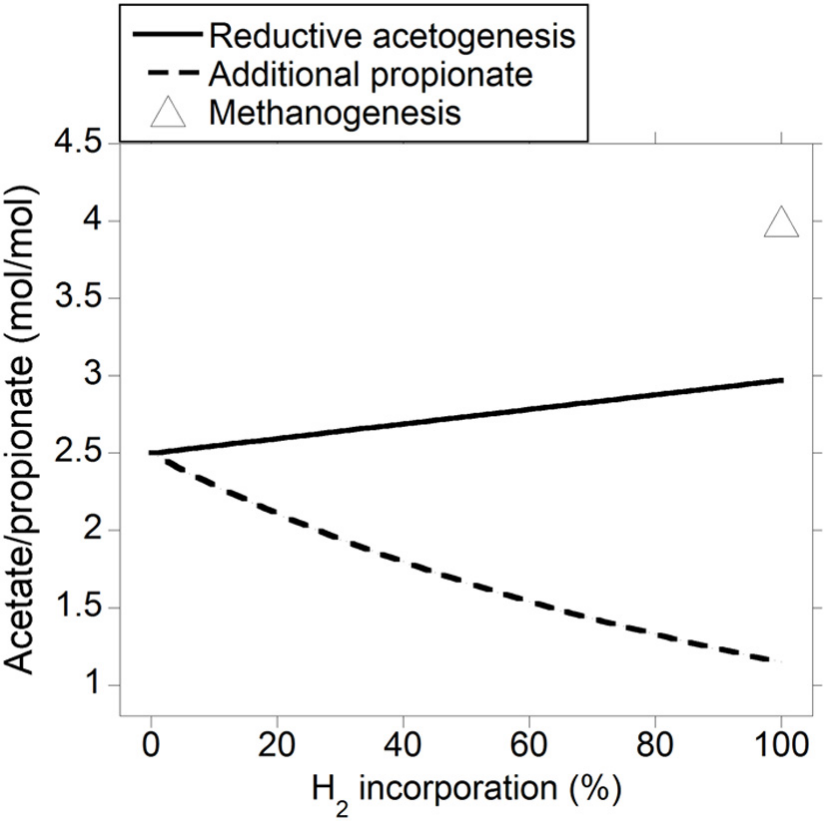
**Response of the acetate to propionate molar ratio to H_2_ incorporation into reductive acetogenesis or additional propionate formation**. Corresponding fermentation with CH_4_ as main electron sink is provided as reference.

**Figure 4:**
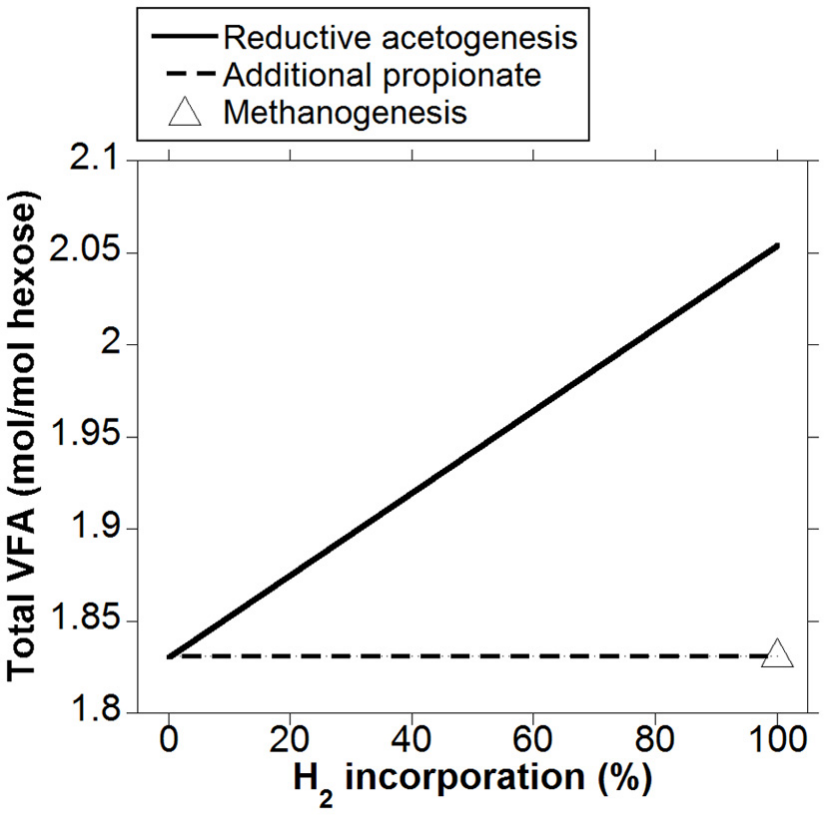
**Response of total VFA production to H_2_ incorporation into reductive acetogenesis or additional propionate formation**. Corresponding fermentation with CH_4_ as main electron sink is provided as reference.

As expected, the acetate to propionate ratio varied in opposite directions as accumulated H_2_ was incorporated into either reductive acetogenesis or additional propionate production, reaching 2.97 and 1.15 for total accumulated H_2_ incorporation into reductive acetogenesis and additional propionate production, respectively, (Figure 3). Hydrogen incorporation into reductive acetogenesis increased total VFA production, whereas H_2_ incorporation into additional propionate production did not change total VFA production because each additional mol of propionate was formed at the expense of one mol of acetate not produced (Figure 4).

**Figure 5:**
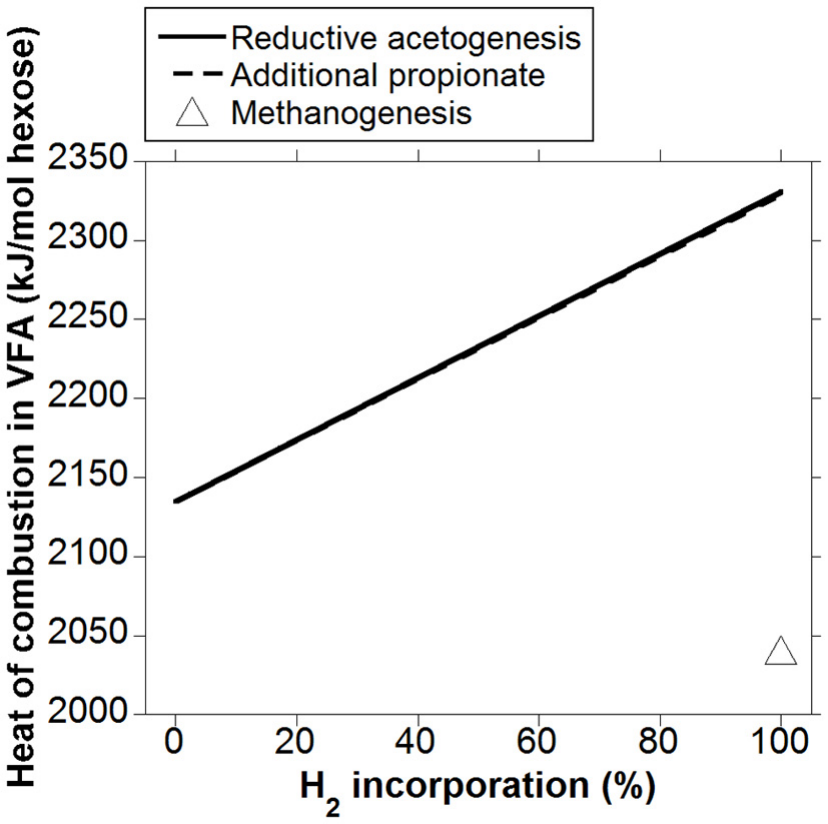
**Response in heat of combustion in VFA to H_2_ incorporation into reductive acetogenesis or additional propionate formation**. Corresponding fermentation with CH_4_ as main electron sink is provided as reference.

Heat (or enthalpy) of combustion is equal to the total energy released as heat by complete oxidation of a compound to CO_2_ and H_2_O at constant pressure. Whereas heat of combustion in gaseous fermentation products like CH_4_ and H_2_ is lost to the atmosphere, VFA are absorbed through the rumen wall and are the main energy source for ruminants. Heat of combustion in VFA is then accounted as metabolizable energy (ME), as gaseous DE (DE in this analysis corresponds to energy in hexoses available for fermentation) losses have been discounted. Volatile fatty acids heats of combustion obtained from Kohn and Boston (2000) were used to calculate how heat of combustion output in VFA per mol of hexose fermented could vary whether accumulated H_2_ was incorporated into reductive acetogenesis or additional propionate (Figure 5). The responses in heat of combustion in VFA were virtually identical for H_2_ incorporation into reductive acetogenesis or additional propionate formation. Total H_2_ incorporation into either process resulted in a 12% gain in heat of combustion output in VFA compared to the corresponding fermentation with CH_4_ as main electron sink.

Even though incorporation of accumulated H_2_ into both reductive acetogenesis and additional propionate formation resulted in less CO_2_ released per mole of fermented hexose (not shown) due to CO_2_ incorporation into acetate or propionate, respectively, CO_2_ molar fraction in total gas produced actually increased (not shown). This is because the number of moles of H_2_ incorporated into either pathway was proportionally greater than the incorporation of CO_2_ [the ratio between H_2_ and CO_2_ incorporated was of 2 to 1 for reductive acetogenesis (Equation 4) and of 3 to 1 for additional propionate formation (Equation 8)], and the small amount of CH_4_ remained constant. The implication is that, if total gas pressure remained constant (i.e., ~10^5^ Pa), CO_2_ pressure would actually increase as H_2_ was incorporated into reductive acetogenesis or additional propionate formation, because greater number of H_2_ moles relative to CO_2_ would be incorporated into either pathway (not shown).

**Figure 6:**
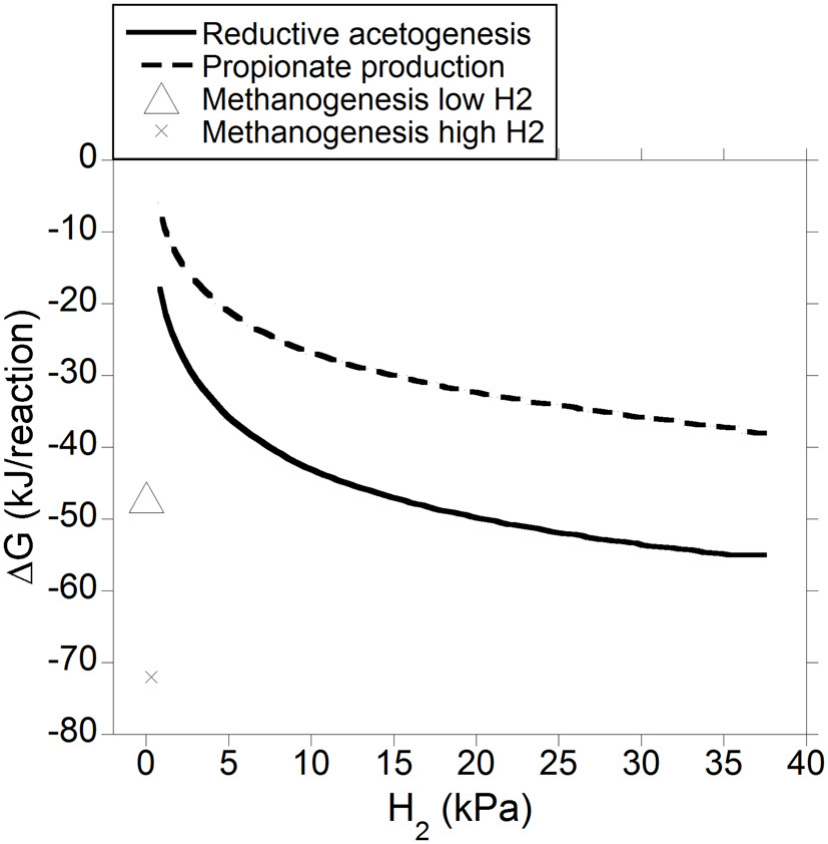
**Estimated ΔG for reductive acetogenesis or additional propionate formation as a function of H_2_ incorporation**. Corresponding fermentation with CH_4_ as main electron sink is provided as reference.

**Figure 7:**
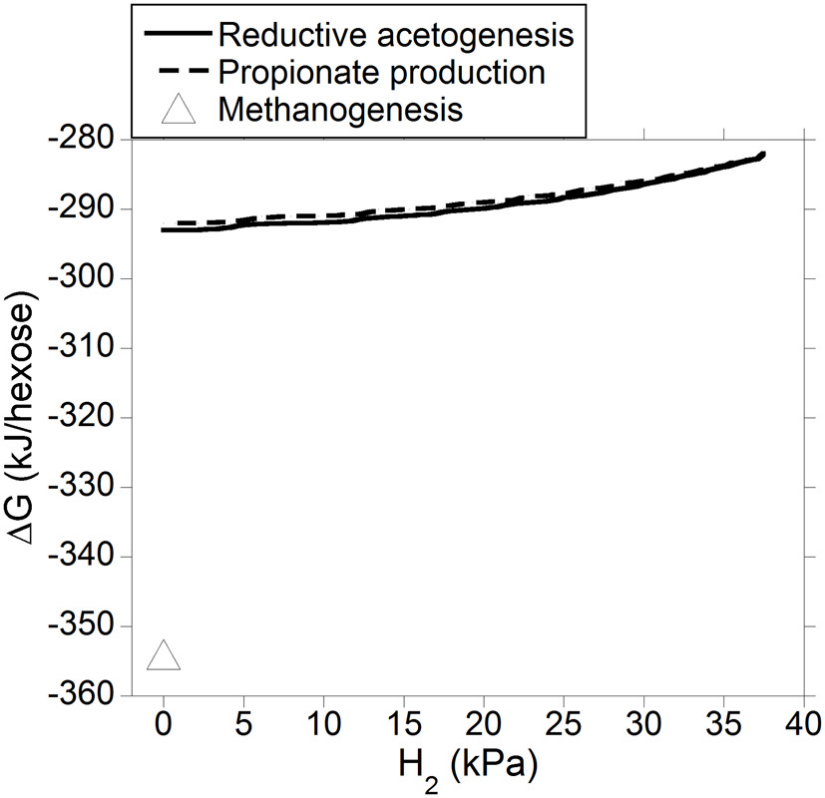
**Estimated ΔG of fermentation with H_2_ incorporation into reductive acetogenesis or additional propionate formation as a function of H_2_ incorporation**. Corresponding fermentation with CH_4_ as main electron sink is provided as reference.

Gibbs energy change (or Gibbs free energy change; ΔG) of a chemical reaction is the theoretical limit to the amount of work that can be extracted from that reaction at constant pressure and temperature; because this limit would theoretically only be reached in a completely reversible reaction, it is never reached in a real non-equilibrium system. Reactions with negative ΔG (exergonic) occur spontaneously provided their activation energy is overcome (catalysts decrease the activation energy of the reaction, allowing it to proceed faster). Reactions with positive ΔG (endergonic) are non-spontaneous i.e., they are thermodynamically unfeasible unless coupled to a reaction with negative ΔG larger in absolute value. Reactions with ΔG equal to zero are at equilibrium and their forward rate is equal to their reverse rate. Gibbs energy change was calculated both for incorporation of accumulated H_2_ into reductive acetogenesis or propionate production (Figure 6), and for the complete fermentation of glucose to VFA and gases for both electron-incorporating processes (Figure 7), as H_2_ incorporation varied between 0 and 100%, assuming a total gas pressure of 10^5^ Pa. Standard ΔG of formation of metabolites in Equation 1, 6 and 10 were obtained from Kohn and Boston (2000) and Karadagli and Rittman (2007). Gibbs energy changes were adjusted to a ruminal temperature of 39°C through the Van't Hoff equation (Kohn and Boston, 2000). Partial pressure for each gas was calculated from the Ideal Gas Law, and dissolved gases concentrations were calculated from Henry's Law (Kohn and Boston, 2000; Janssen, 2010). Glucose concentration was assumed to be 6 × 10^−4^ M (Janssen, 2010). Water concentration was assumed to be 50 M (Kohn and Boston, 2000). It is acknowledged that the upper limit of H_2_ pressure in Figure 6 is high for most methanogenesis-inhibition experiments, but similar values have been reported in some *in vitro* batch culture (Van Nevel et al., 1969; O'Brien et al., 2013) and *in vivo* (Rufener and Wolin, 1968; Trei et al., 1971, 1972; Kung et al., 2003) experiments where methanogenesis was inhibited.

Figure 6 depicts the thermodynamic feasibility of further incorporation of accumulated H_2_ into reductive acetogenesis (Equation 4) or propionate formation from acetate, CO_2_ and H_2_ (Equation 8) with varying H_2_ pressure resulting from different percentages of accumulated H_2_ incorporation into either pathway. Gibbs energy change of methanogenesis corresponding to a low and a high H_2_ concentration of 0.2 and 2.3 μM, respectively, is shown as reference (low and high H_2_ concentration values for high forage diets were obtained from Janssen, 2010, and converted to pressure using Henry's Law). As H_2_ pressure approached typical ruminal levels, both reductive acetogenesis and conversion of acetate to propionate rapidly approached equilibrium, as described by Ungerfeld and Kohn (2006), whereas, expectedly, methanogenesis was still thermodynamically favorable (Kohn and Boston, 2000).

Figure 7 shows how the entire fermentation ΔG responded to varying H_2_ pressure resulting from accumulated H_2_ incorporation into reductive acetogenesis (Equation 6) or additional propionate formation (Equation 10), along with the corresponding roughage fermentation with CH_4_ as main electron sink (Equation 1). Estimated ΔG was comparable for both fermentation processes and varied little as accumulated H_2_ was incorporated into either reductive acetogenesis or additional propionate formation. Fermentation with CH_4_ as main electron sink was more favorable than theoretical 100% incorporation of accumulated H_2_ into either reductive acetogenesis or additional propionate formation.

## Discussion

Consequences of incorporation of accumulated H_2_ resulting from methanogenesis inhibition into reductive acetogenesis or additional propionate will be discussed with respect to four aspects of ruminant nutrition interest: fermentation energetics, microbial protein production, ruminal pH, and VFA post-absorptive metabolism.

### Fermentation energetics

**Figure 8:**
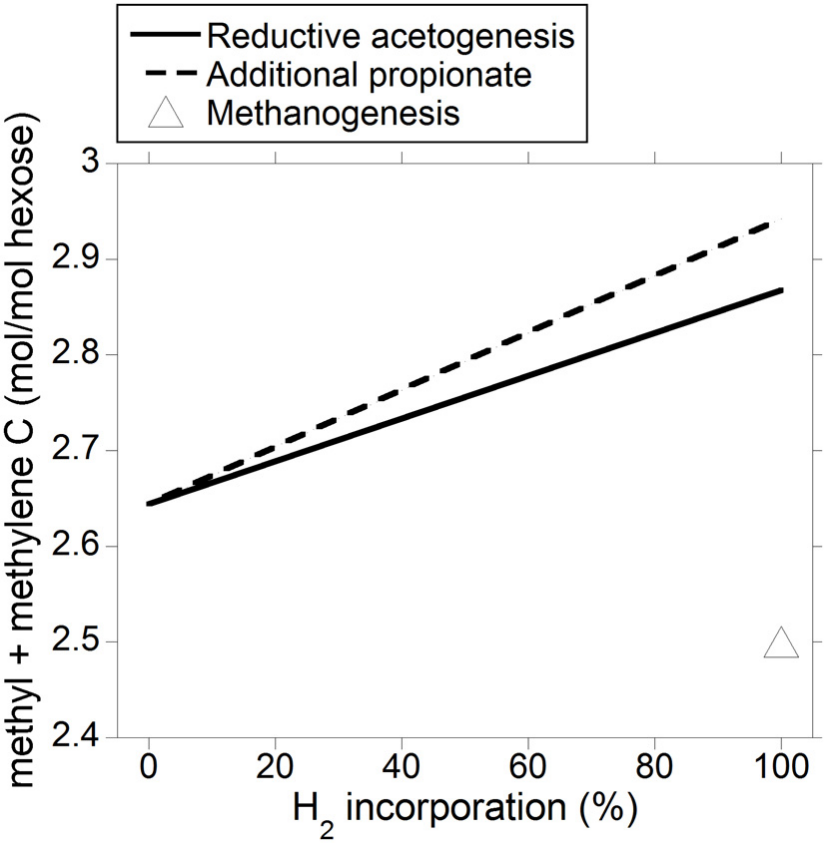
**Response in total VFA methyl plus methylene groups output to H_2_ incorporation into reductive acetogenesis or additional propionate formation**. Corresponding fermentation with CH_4_ as main electron sink is provided as reference.

The energetic equivalence of incorporating accumulated H_2_ into reductive acetogenesis or additional propionate is a result of the stoichiometries of both processes and of acetate and propionate heats of combustion. Reduction of 2 mol of CO_2_ with 4 mol of H_2_ to produce 1 mol of acetate results in a gain in heat of combustion in acetate per electron-pair incorporated equal to 876 kJ/4 mol H_2_ = 219 kJ/mol H_2_. Incorporation of 3 mol of H_2_ to produce 1 mol of propionate at the expense of 1 mol of acetate not being produced (Equation 9) results in a gain in heat of combustion in propionate per electron-pair incorporated equal to: (1529–876 kJ)/3 mol H_2_ = 218 kJ/mol H_2_. Ultimately, this result is predicted by the balance of the different types of chemical bonds broken and made (calculations not shown); even though incorporating accumulated H_2_ into reductive acetogenesis would result in greater total C retention in VFA compared to propionate production (not shown), a mole of propionate contains twice as many C atoms moles susceptible to be oxidized (i.e., methyl and methylene groups) than a mole of acetate, and total methyl plus methylene groups formed would be actually slightly greater if accumulated H_2_ was incorporated into propionate (Figure 8).

Incorporation of H_2_ into either reductive acetogenesis or propionate production resulted in an increase in heat of combustion in VFA of about 12% with respect to the corresponding fermentation with CH_4_ as main electron sink. As discussed, this energy gain corresponds to ME. Given that a range of 2 to 12% gross energy is lost as CH_4_ (Attwood and McSweeney, 2008), the corresponding range for a roughage diet of, for example, 60% energy metabolisability, would be 3 to 20%. Therefore, the proportion of ME lost as CH_4_ agrees with previous sources (Attwood and McSweeney, 2008).

Apart from what could be the energetic consequence of incorporating accumulated H_2_ into reductive acetogenesis or propionogenesis, a very important question is, of course, why neither process is the main electron sink in ruminal fermentation, and methanogenesis is instead the dominant electron sink.. It has been shown that reductive acetogenesis is negligible in ruminal incubations (Nollet et al., 1997a; Le Van et al., 1998), and attempts to induce it by adding reductive acetogens have been unsuccessful (López et al., 1999). Reductive acetogens have been isolated from the rumen (Leedle and Greening, 1988) and identified using molecular techniques (Henderson et al., 2010), but they seem to rely on mixotrophic growth i.e., substrates other than CO_2_ and H_2_, and to use diverse electron acceptors (Joblin, 1999; Drake et al., 2002).

As a redox process, methanogenesis has a lower H_2_ threshold than reductive acetogenesis (Cord-Ruwisch et al., 1988), which allows methanogens to lower H_2_ partial pressure at a level at which reductive acetogenesis is thermodynamically unfeasible; reductive acetogenesis has been estimated to be close to equilibrium at ruminal conditions even without considering ATP generation (Kohn and Boston, 2000). Thus, adding reductive acetogens to rumen fermentation only enhances reductive acetogenesis enzyme kinetics, whereas the process is thermodynamically limited and therefore methanogenesis remains dominating. Two different low H_2_-threshold acetogens decreased methanogenesis between 64 and 97% when co-cultured with *Methanobrevibacter* sp. (Joblin, 1999); however, these co-cultures were grown under elevated H_2_ pressure, which would remove methanogenesis thermodynamic advantage, as opposed to ruminal conditions, where H_2_ originates only from fermentation. This experiment though would demonstrate a kinetic advantage in terms of *V*_max_ for utilizing H_2_ of these acetogens over their *Methanobrevibacter* competitor when reductive acetogenesis thermodynamic disadvantage was overcome. It is suggested to repeat this kind of experiments both in the presence of external H_2_ and as tri-cultures with H_2_-producing organisms. Similarly, the presence of a live yeast enhanced the competitive capacity of a reductive acetogen against a methanogen (Chaucheyras et al., 1995), but this again happened when the thermodynamic constraint to reductive acetogenesis was removed by growing the tri-culture under artificially elevated H_2_ pressure.

Whereas it does not occur in the rumen, reductive acetogenesis co-exists with or even dominates over methanogenesis in other gut environments: wood-digesting termites, rodents, pigs, humans (Joblin, 1999), young rabbits (Piattoni et al., 1996), marsupials (Ouwerkerk et al., 2009), ostriches (Fievez et al., 2001), newborn lambs (Morvan et al., 1994) and ruminant hindguts (Immig, 1996), contributing to the host animal energetic requirements. Addition of the reductive acetogen *Peptostreptococcus productus* ATCC35244 to an *in vitro* reactor simulating the human gut inoculated with human faces resulted in a decrease in methanogenesis to a level below detection limit (Nollet et al., 1997b), but did not affect methanogenesis in ruminal incubations (Nollet et al., 1997a). It is therefore important to understand the reasons for why reductive acetogenesis occurs in those environments and does not occur in the rumen.

Despite methanogenesis being thermodynamically more favorable than reductive acetogenesis, conditions like low pH or low temperature can diminish methanogenesis and make reductive acetogenesis more competitive in certain freshwater lake sediments, flooded rice paddies or tundra wetland soils (Drake et al., 2002). Transiently elevated H_2_ concentration in marine sediments resulting from a low sulphate concentration period allowed reductive acetogenesis to become thermodynamically feasible (Hoehler et al., 1999). In the wood-feeding termite *Reticulitermes flavipes* hindgut, reductive acetogens occupy the more reduced lumen with high H_2_ pressure, and methanogens are displaced to the micro-oxic periphery (Ebert and Brune, 1997). Association of reductive acetogens to H_2_-producing protozoa in the termite gut also seems to contribute to favoring reductive acetogenesis over methanogenesis in that environment (Leadbetter et al., 1999). In agreement, reductive acetogenesis seems to be an important hydrogen sink in the ruminant hindgut, where protozoa are absent (Immig, 1996). However, an explanation is yet missing for why spatial differences that may allow reductive acetogenesis becoming thermodynamically feasible in wood-feeding termite hindguts do not occur in the rumen i.e., why ruminal reductive acetogens do not displace methanogens from the more reducing microniches and why they do not associate with protozoa. Perhaps, rates of H_2_ production in environments like the wood-feeding termites hindgut lumen are greater than in the rumen and remove thermodynamic constraints to reductive acetogenesis. Under those conditions reductive acetogenesis could become dominant if reductive acetogens had a greater capacity to utilize H_2_ at high concentration (i.e., higher *V*_max_) than methanogens; then, as H_2_ availability decreases toward the termite hindgut periphery, reductive acetogenesis would become thermodynamically unfeasible and methanogenesis would become the H_2_ sink close to the hindgut epithelium. It is also possible that at low H_2_ pressure methanogens rely on a lower *K*_m_ for H_2_ to establish in the termite gut periphery. Although I am not aware of measured kinetic parameters of methanogens and reductive acetogens competing for H_2_ in the same environment, it has been proposed that microbial specialists (methanogens in this case) have greater affinities for the substrates they compete for than their generalist competitors (Lever, 2012).

As opposed to reductive acetogenesis, propionate production is already an electron sink in ruminal fermentation, but second to methanogenesis with regard to its quantitative importance. As it has been shown, CH_4_ production and the VFA profile are intimately related. From a purely stoichiometric point of view, propionate could, theoretically, replace CH_4_ as an electron sink. For example:

(11) $$\begin{array}{l} {\text{C}}_{6}{\text{H}}_{12}{\text{O}}_{6}\to 2/3\;{\text{CH}}_{3}{\text{COO}}^{-}+4/3\;{\text{CH}}_{3}{\text{CH}}_{2}{\text{COO}}^{-}+2\;{\text{H}}^{+}\\ \;+2/3\;{\text{CO}}_{2}+2/3\;{\text{H}}_{2}\text{O} \end{array}$$

It is clear, however, that the stoichiometry proposed in Equation 11 is well outside what is observed for ruminal fermentation. The question that then arises is: is the competition between methanogenesis and propionate production for metabolic hydrogen in the rumen controlled by thermodynamics or by affinity for H_2_? Co-culturing of succinate and propionate producers with methanogens resulted in a decrease in succinate or propionate production (Chen and Wolin, 1977; Latham and Wolin, 1977; Marvin-Sikkema et al., 1990). In my opinion, those shifts in fermentation in co-cultures cannot not be explained by affinity for H_2_, because H_2_ transferred to methanogens needs to be first produced by hydrogenases in the fermentative organism; any competition for H_2_ must have been preceded by an intracellular shift in the flux of metabolic hydrogen driven either by thermodynamics or by kinetic competition between hydrogenases and fumarate or acrylyl-CoA reductases for NADH.

Of course, the above results with co-cultures apply to organisms possessing the metabolic capacity to produce propionate or succinate, but not necessarily to the competition for H_2_ produced by the mixed microbiota that does not have this capacity. At least some propionate producers are able to use H_2_ to reduce fumarate to succinate (Henderson, 1980), which is manifested by the shift from acetate to propionate production occurring with an increase in H_2_ pressure (Janssen, 2010). How is then the competition between methanogens and propionate producers for the extracellular H_2_ pool controlled? It has been shown that methanogens have a lower *K*_m_ for H_2_ than fumarate reducers (Russell and Wallace, 1997; Asanuma et al., 1999). On the other hand, an analysis derived from published values concluded that opposite interconversion flows between VFA (i.e., Equation 8) are generally similar, which was interpreted as an indication that VFA are close to equilibrium much of the time, and that the range of VFA ratios commonly observed for ruminal fermentation, and hence CH_4_ production, could be the result of thermodynamic control (Ungerfeld and Kohn, 2006).

Another important problem is that, as discussed, even in the methanogenesis-inhibited ruminal fermentation, neither reductive acetogenesis nor propionate production, incorporate the totality of reducing equivalents from inhibited CH_4_, as manifested by the fact that H_2_ accumulates. The question, therefore, is why H_2_ accumulates instead of being incorporated into reductive acetogenesis or propionate production or both. Organisms conducting the pathways depicted en Equation 4 and 8 would need to utilize some of the negative ΔG associated to those reactions to generate ATP to make their own endergonic anabolic reactions thermodynamically feasible to grow fast enough so as to not get washed out of the rumen. Gibbs energy change of ATP hydrolysis to ADP and phosphate is about −50 kJ/mol (Voet and Voet, 1995). Then, the ΔG needed for the reverse reaction, the generation of 1 mol of ATP, appears to be similar to the ΔG of reductive acetogenesis (Equation 4) and propionate production from acetate, CO_2_ and H_2_ (Equation 8) at 37 kPa H_2_ (0% incorporation of accumulated H_2_; Figure 6). This may therefore be taken as an indication that H_2_ accumulation in the methanogenesis-inhibited rumen results from the system reaching thermodynamic equilibrium regarding further incorporation of H_2_ reducing equivalents into reductive acetogenesis or propionate production. However, this interpretation would conflict with the decrease in H_2_ pressure observed upon addition of reductive acetogens to methanogenesis-inhibited batch cultures (Nollet et al., 1997a; Le Van et al., 1998; López et al., 1999). Yet, organisms growing in batch cultures do not get washed out, and perhaps they could survive generating less than 1 ATP mol through ion gradient-driven phosphorylation per mol of acetate (Equation 4) or propionate (Equation 8) produced. Also, the discrepancy between the thermodynamic calculation for reductive acetogenesis in Figure 6 and the experimental *in vitro* results (Nollet et al., 1997a; Le Van et al., 1998; López et al., 1999) could be explained by H_2_ oversaturation in the liquid phase, where it is generated, with respect to the gas phase, which would result in H_2_-utilizing reactions being more favorable for microbes than what calculations assuming H_2_ saturation would suggest (Hackmann, 2013). Furthermore, H_2_ gradients exist in anaerobic environments (Boone et al., 1989), and are likely to exist between liquid and particle ruminal compartments (Czerkawski, 1986), making some reactions more favorable at particular locations. An example of a ruminal H_2_ gradient is the physical association of some methanogens with protozoa to be closer to a H_2_ source (Morgavi et al., 2010).

Through adding propionate formation intermediates, Tatsuoka et al. (2008) and Ebrahimi et al. (2011) had some success at decreasing H_2_ accumulation in methanogenesis-inhibited ruminal batch incubations, which would indicate, that at least under their conditions, propionate formation was limited by thermodynamics or substrate kinetics (through the availability of a C skeleton). Fonty et al. (2007) found that addition of fumarate to a methanogen-free ruminal incubation accelerated H_2_ utilization without affecting its final point at 24 h, which would suggest a limitation of a substrate kinetic nature.

### Microbial protein production

With most ruminant diets, microbial protein is the principal source of amino acids for the host animal (Bach et al., 2005). It is also the cheapest one, as ruminants can use N sources with no nutritional value to non-ruminants (Wallace et al., 1997). Microbial protein synthesis can be understood as the product between the number of moles of ATP generated in fermentation and the amount of protein synthesized per mole of ATP hydrolyzed.

Generation of ATP is made possible by negative ΔG of fermentation reactions. Fermentation ΔG for incorporation of accumulated H_2_ into reductive acetogenesis or additional propionate formation was estimated to be similar (Figure 7). However, only part of fermentation ΔG in Figure 7 is available to generate ATP; if all of ΔG associated to fermentation was coupled to an endergonic process like ATP generation, fermentation would be at equilibrium and come to a halt. It is difficult to compare Equation 6 and 10 with regard to ATP generation. Generation of ATP associated to ion-gradient-driven phosphorylation occurs both in reductive acetogenesis (Müller, 2003), the propionate randomizing pathway (Russell and Wallace, 1997), and methanogenesis (Thauer et al., 2008), and the stoichiometry of ATP generation per pair of electrons may not be constant (Reddy and Peck, 1978; Kröger and Winkler, 1981; Thauer et al., 2008). In addition, ATP generation in propionate production would vary depending on the mechanism of oxaloacetate formation and the ratio of C flow through the randomizing (via oxaloacetate and succinate) vs. non-randomizing (via lactate) pathway (Russell and Wallace, 1997). Thus, the proportion of fermentation ΔG in Figure 7 actually utilized by cells to generate ATP is unknown and could vary depending on the organism and conditions.

The typical roughage fermentation with CH_4_ as main electron sink depicted in Equation 1 appeared to be more exergonic than either Equation 6 or Equation 10 at any point of H_2_ accumulation (Figure 7), and, perhaps could conserve greater ΔG per mol of glucose fermented. On the other hand, methanogenesis inhibition *in vitro* has resulted in an increase in microbial N production (Ungerfeld et al., 2007; Guo et al., 2009), possibly due to greater availability of reducing equivalents for anabolic reactions (Czerkawski, 1986). Thus, even if methanogenesis inhibition resulted in a less exergonic fermentation, production of microbial N protein might still be favored because synthetic reactions could become energetically less costly (Czerkawski, 1986). More experimentation is needed to understand the effects of methanogenesis inhibition on microbial growth, and alternative explanations to stimulation of anabolic reactions have been proposed. Ungerfeld et al. (2007) also speculated that the methanogenesis inhibitor propynoic acid might have improved microbial growth and efficiency through protozoal inhibition, although protozoa were not quantified in that study. Lila et al. (2004) and Mohammed et al. (2004) did not find changes in protozoal numbers as a consequence of inhibiting CH_4_ production in ruminal batch incubations. Microbial biomass production was not measured in those studies.

The second factor determining the microbial protein production is the amount of protein synthesized per mole of ATP hydrolyzed. Anabolic efficiency is influenced by factors such as the presence of glucose phosphotransferase transport systems, disaccharides phosphorylases, energy conservation through transmembrane electrochemical gradients, variation in cell composition, variation in maintenance energy, and energy-spilling reactions (Russell and Wallace, 1997). It is unknown how mixed microbial populations conducting the pathways depicted in Equation 6 and 10 could compare with regards to those factors. Because they use the reductive acetyl-CoA pathway for both C fixation and catabolism, it has been speculated that reductive acetogens might be able to survive and replicate maintaining smaller genomes and fewer enzymes, which could decrease their energy requirements (Lever, 2012). Experimentation would be needed to understand how incorporation of accumulated H_2_ into reductive acetogenesis or propionate production can affect microbial protein production.

### Ruminal pH

Incorporation of accumulated H_2_ into reductive acetogenesis would result in increased total VFA production, whereas additional propionate formation would not affect it (Figure 4). The relationship between VFA concentration and pH was weak across studies with different diets (Allen, 1997), but for this analysis a common basal diet is assumed, and possible changes in ruminal pH are discussed as the sole consequence of H_2_ incorporation into reductive acetogenesis or propionate production. Changes in VFA concentration need not reflect changes in VFA production proportionally, as VFA absorption also increases (Penner et al., 2009b, 2011); therefore, the calculation that follows represents an upper limit to pH decrease as a consequence of greater VFA production.

Assuming a hypothetical but realistic total VFA concentration of 100 mM for maximum H_2_ accumulation (Equation 3), total incorporation of accumulated H_2_ into reductive acetogenesis (Equation 5) could result in a total VFA concentration of 112 mM due to maximal additional 12 mM acetate (calculation from Figure 4), if greater VFA production was not compensated to any extent by greater absorption. Buffering capacity (BC) of a solution is the molar H^+^ concentration required to cause a change in pH, at a certain pH: BC = *d*H^+^/*d*pH (Counotte et al., 1979). Ruminal fluid BC reported in various studies is summarized in Table 1. Then, the expected decrease in pH for the maximum extra 12 mM H^+^ due to an additional 12 mM acetate concentration would be equal to −0.012 M/-BC.

**Table 1:** **Ruminal fluid buffering capacity determinations and estimated maximal pH decrease for H_2_ incorporation into reductive acetogenesis**.

**Reference**	**BC (average and range, M H^+^/pH unit)**	**pH of BC determination**	**Calculated pH decrease**
Turner and Hodgetts (1955)^a^	0.028 (0.013–0.042)	5.80–6.90	0.63
Hodgson and Thomas (1975)	0.071 (0.062–0.076)	5.54–6.84	0.37
Counotte et al. (1979)^b^	0.038 (0.021–0.063)	6.2^c^	0.52
Murphy et al. (1983)^d^	0.013 (0.009–0.02)	7^e^	1.12
Tissera et al. (1988)	0.037 (0.037–0.038)	5.5 to 5.0^f^	0.52
Froetschel and Amos (1991)^b^	0.046 (0.044–0.048)	6.2^g^	0.46

a Determined under a CO_2_ and N_2_ mixture (proportions not reported).

b Determined under CO_2_.

c Ruminal fluid pH was 6.09–7.20.

d Determined under a 50:50 CO_2_:N_2_ mixture and a 5 mm layer of mineral oil.

e Ruminal fluid pH was 5.80–6.14.

f Ruminal fluid pH was 5.77–5.81.

g Ruminal fluid pH was 6.49–6.8.

In addition, if total gas pressure remained at 1 atm after total incorporation of accumulated H_2_ into reductive acetogenesis or additional propionate, CO_2_ pressure would increase from approximately 0.60 to 0.95 atm (calculations not shown). Based on Kohn and Dunlap (1998), it can be derived (not shown) that if bicarbonate concentration remained constant, pH would change by:

(12) ΔpH=log(0.60atm/0.95atm)=−0.20

The expected decrease in pH for H_2_ incorporation into reductive acetogenesis calculated from BC, adjusted for Equation 12, is shown in Table 1. In most cases, there was a moderate pH decrease. It should be considered that changes in VFA production could affect other physiological mechanisms such as buffer secretion due to bicarbonate secreted by rumen epithelium in exchange for absorbed VFA anions, most importantly acetate (Penner et al., 2009a; Aschenbach et al., 2011). Therefore, effects of H_2_ incorporation into reductive acetogenesis or additional propionate on ruminal pH are difficult to predict accurately, and *in vivo* experimentation would be needed to completely understand how ruminal pH could be affected.

### Volatile fatty acids post-absorptive metabolism

#### Efficiency of VFA utilization

Utilization by the animal of heat of combustion in VFA is incomplete, as heat is lost in the conversion of ME to net energy (NE) for maintenance or production (heat increment, HI). Initial experiments with intra-ruminally-infused VFA in fasting (Armstrong and Blaxter, 1957a; Armstrong et al., 1957) and fattening sheep (Armstrong and Blaxter, 1957b) resulted in greater HI for pure acetate compared to pure propionate. Mixtures of VFA were similar to pure propionate in fasting animals (Armstrong and Blaxter, 1957a; Armstrong et al., 1957), but a VFA mixture high in acetate was associated to a greater HI compared to one high in propionate when administered to sheep fed above maintenance (Armstrong et al., 1958). Later work found similar HI for VFA mixtures varying in acetate and propionate proportions in lactating cows (Ørskov et al., 1969), fasting and fattening lambs (Ørskov et al., 1979), and steers at different feeding levels, except for a decreased HI at the highest acetate molar proportion due to acetate excretion in urine (Ørskov et al., 1991; Ørskov and MacLeod, 1993).

Therefore, it seems that the differences in VFA profiles resulting from the incorporation of accumulated H_2_ into reductive acetogenesis or additional propionate formation may not affect the efficiency of absorbed VFA utilization in animals at maintenance, thus likely resulting in similar NE outputs, given that heat of combustion output in VFA was similar (Figure 5); this issue would be unresolved for producing animals.

#### Post-absorptive metabolic effects of VFA

Apart from being energy sources, absorbed acetate and propionate have different metabolic consequences for ruminant post-absorptive metabolism (DiConstanzo et al., 1999). As a general frame of analysis, one could conceive the extra acetate or propionate formed through incorporation of accumulated H_2_ as an intervention analogous to adding a ketogenic or glycogenic supplement, respectively, to a basal diet. Although dietary effects on energy partition and milk production and composition are primarily explained by duodenal flow of conjugated linoleic acid isomers, they also partly respond to changes in production of acetate and propionate in the rumen (Maxin et al., 2011). It should be cautioned though that changes in pH and individual VFA ruminal concentrations could affect individual VFA absorption fluxes differently: absorption flux of propionate, but not of acetate, increased as a result of greater inclusion of dietary concentrates and corresponding greater propionate molar proportion and lower pH; the effect of diet on each VFA absorption flux seemed to follow changes in their concentrations, as rumen volume and fractional absorption rates of individual VFA were unaffected (Penner et al., 2009b).

Propionate is the main glucose precursor in ruminants, so it may be advantageous to produce more propionate in animals with high glucose requirements e.g., high-producing dairy cows (Aschenbach et al., 2010). However, dairy cows fed a ketogenic diet partitioned more energy to milk compared to those fed a glycogenic diet equal in NE (Van Knegsel et al., 2007a,b,c), which agrees with changes in energy excretion in milk observed as a response to intra-ruminal infusion of acetate or propionate (Ørskov et al., 1969) and intramesenteric infusion of propionate (Casse et al., 1994). Ketogenic diets generally resulted in greater milk fat content and production, whereas both were lesser with glycogenic diets (Van Knegsel et al., 2005, 2007a,b). In agreement, intra-ruminal infusion of acetate resulted in greater milk fat content and production compared to propionate infusion (Ørskov et al., 1969; Sheperd and Combs, 1998; Maxin et al., 2011). On the contrary, glycogenic diets often increased, and ketogenic diets decreased, milk protein content (Van Knegsel et al., 2005, 2007a,b). Propionate infusion resulted in greater milk protein content (Sheperd and Combs, 1998).

However, ketogenic diets are associated to greater negative energy balance and risk of ketosis (Van Knegsel et al., 2005, 2007a,c). Importantly, there can be an interaction between the basal diet and the supplement regarding the risk of ketosis. Supplementing a ketogenic basal diet with fat resulted in an increase in circulating concentration of ß-hydroxybutyrate, in contrast to a glycogenic basal diet (Van Knegsel et al., 2005). Thus, consequences of incorporating accumulated H_2_ into reductive acetogenesis or additional propionate on the risk of ketosis may be more important with forage-based and fat-supplemented diets than with mixed diets.

Van Knegsel et al. (2005) did a thorough analysis of studies on the effects of ketogenic or glycogenic supplementation on reproduction and found it difficult to conclude on consistent effects. However, direct comparisons between ketogenic and glycogenic diets suggested that the latter may be advantageous for reproduction (Van Knegsel et al., 2007a,c).

In animals with a production potential not limited by glucose supply, or with no risk of ketosis, there may be no a clear advantage of reductive acetogenesis or propionate formation as electron-incorporating pathway. Moreover, propionate is a major satiety signal in ruminants (Allen et al., 2009); thus, it may be preferable to incorporate accumulated H_2_ into reductive acetogenesis in animals whose basal diet already satisfies their glucose requirements, if their dry matter intake (DMI) is metabolically constrained, e.g., steers on high-concentrate diets. However, in feedlot steers with subclinical acidosis, incorporating H_2_ into reductive acetogenesis could result in further pH decrease, which could in turn diminish DMI. There may not be advantages to reductive acetogenesis vs. additional propionate formation as an electron-incorporating pathway in animals with low glucose requirements if feed intake is not to be maximized e.g., beef cows.

## Future directions

Evidently the conclusions reached through the present analysis would have to be tested through experimentation. Experimentally comparing effects of redirecting methanogenesis electrons toward reductive acetogenesis or propionate production on animal physiology and production is at present difficult, because attempts to inhibit methanogenesis have had little success in incorporating electrons into one or the other pathway. In my opinion, a question that precedes the question on how to incorporate methanogenesis electrons into reductive acetogenesis or propionate production, is, what limits those processes in the rumen i.e., how is H_2_ incorporation into reductive acetogenesis and propionate production physico-chemically controlled. Physico-chemical control of H_2_ incorporation into reductive acetogenesis or propionate production could be based primarily on enzyme or substrate kinetics, or thermodynamics. It is important to understand this in order to efficiently design strategies that can achieve successful incorporation of methanogenesis electrons into these pathways.

For example, an enzyme kinetics limitation could be solved through microbial intervention e.g., microbial additives, overexpression of particular genes etc. On the other hand, a limitation of a substrate kinetics or thermodynamics nature would require an intervention at the level of substrate availability and/or product removal, and might be less feasible or profitable in practice.

It would be best to test these hypotheses in very simplified systems, like chemostats running on soluble substrates where steady state constant kinetics and thermodynamic conditions can be attained. Defined cultures experiments on interspecies H_2_ transfer similar to the ones conducted some decades ago but including measurements of changes in the expression of genes encoding key enzymes such as hydrogenases and fumarate and acrylyl-CoA reductases, as well as the activities of those enzymes, are also suggested. Once this basic understanding was achieved, experimentation could move toward more realistic *in vitro* systems using solid substrates, and finally *in vivo* conditions. The underlying principle beyond this proposal is that a system can be better manipulated if we can understand how the flow of metabolites is controlled. Mechanistic understanding of ruminal fermentation will provide a long term solid basis to develop efficient strategies of manipulation of rumen fermentation and better predict and understand the outcomes of new strategies.

In summary, incorporating accumulated H_2_ into reductive acetogenesis or additional propionate formation was found to be equivalent in terms of ME, and of NE for animals at maintenance; whether the efficiency of conversion of ME to NE in producing animals could be different if H_2_ was incorporated into reductive acetogenesis or additional propionate formation would remain unresolved. Incorporation of accumulated H_2_ into reductive acetogenesis could result in moderate ruminal pH decrease, although *in vivo* experimentation would be needed to characterize complex whole animal responses, as whole-animal responses other than ruminal fermentation could be affected. Research would be needed on the consequences of H_2_ incorporation into either pathway on microbial protein production. Post-absorptive consequences of either pathway on energy partition into milk and body tissues, risk of ketosis in dairy cows, and DMI could differ. Utilization of reductive acetogenesis as the preferred electron-incorporating pathway could stimulate energy partition into milk over body fat, although in high-producing dairy cows eating ketogenic diets could result in greater risk of ketosis. Incorporating accumulated H_2_ into propionate could increase milk protein and could decrease the risk of ketosis in animals eating ketogenic diets. However, in animals with metabolically constrained DMI and sufficient propionate or glucose supply, like steers on high-concentrate diets, incorporation of accumulated H_2_ into propionate could result in decreased DMI; in steers with subclinical acidosis though, reductive acetogenesis may cause further pH decline.

Results of this comparison can be used to guide future research efforts in this area. This analysis shows that the production outcome of a successful ruminal methanogenesis intervention could depend on the alternative electron-incorporating pathway. Because of the different nutritional implications of either pathway, and because practical solutions to incorporate accumulated H_2_ into either pathway are not yet available, it is recommended to continue exploring both reductive acetogenesis and additional propionate production as electron sinks alternative to methanogenesis.

### Conflict of interest statement

The author declares that the research was conducted in the absence of any commercial or financial relationships that could be construed as a potential conflict of interest.

## Appendix A

Summary of equations with decimal coefficients

Equation 1: Example of typical fermentation of glucose with a high-roughage diet

$$\begin{array}{l} {\text{C}}_{6}{\text{H}}_{12}{\text{O}}_{6}\to 1.333\;{\text{CH}}_{3}{\text{COO}}^{-}+0.333\;{\text{CH}}_{3}{\text{CH}}_{2}{\text{COO}}^{-}\\ \;+0.167\;{\text{CH}}_{3}{\text{CH}}_{2}{\text{CH}}_{2}{\text{COO}}^{-}+1.833\;{\text{H}}^{+}+{\text{CO}}_{2}\\ \;+0.667\;{\text{CH}}_{4}+0.333\;{\text{H}}_{2}\text{O} \end{array}$$

Equation 2: Example of typical fermentation of glucose with a high-concentrate diet

$$\begin{array}{l} {\text{C}}_{6}{\text{H}}_{12}{\text{O}}_{6}\to 0.923\;{\text{CH}}_{3}{\text{COO}}^{-}+0.615\;{\text{CH}}_{3}{\text{CH}}_{2}{\text{COO}}^{-}\\ \;+0.231\;{\text{CH}}_{3}{\text{CH}}_{2}{\text{CH}}_{2}{\text{COO}}^{-}+1.769\;{\text{H}}^{+}+0.962\;{\text{CO}}_{2}\\ \;+0.423\;{\text{CH}}_{4}+0.538\;{\text{H}}_{2}\text{O} \end{array}$$

Equation 3: Example of methanogenesis inhibition in high-roughage diet of Equation 1

$$\begin{array}{l} {\text{C}}_{6}{\text{H}}_{12}{\text{O}}_{6}+0.6\;{\text{H}}_{2}\text{O}+1.119\text{ X}\to 1.186{\text{CH}}_{3}{\text{COO}}^{-}\\ \;+0.475\;{\text{CH}}_{3}{\text{CH}}_{2}{\text{COO}}^{-}+0.169\;{\text{CH}}_{3}{\text{CH}}_{2}{\text{CH}}_{2}{\text{COO}}^{-}\\ \;+1.831\;{\text{H}}^{+}+1.469\;{\text{CO}}_{2}+0.895\;{\text{H}}_{2}+0.0559\;{\text{CH}}_{4}\\ \;+1.119\text{X}[2\text{H}] \end{array}$$

Equation 4: Reductive acetogenesis

$$2\;{\text{CO}}_{2}+4{\text{H}}_{2}\to {\text{CH}}_{3}{\text{COO}}^{-}+{\text{H}}^{+}+2{\text{H}}_{2}\text{O}$$

Equation 5: Incorporation of 100% H_2_ in Equation 3 into reductive acetogenesis

$$\begin{array}{l} {\text{C}}_{6}{\text{H}}_{12}{\text{O}}_{6}+0.153{\text{H}}_{2}\text{O}+1.119\text{X}\to 1.410{\text{CH}}_{3}{\text{COO}}^{-}\\ \;+0.475{\text{CH}}_{3}{\text{CH}}_{2}{\text{COO}}^{-}+0.169{\text{CH}}_{3}{\text{CH}}_{2}{\text{CH}}_{2}{\text{COO}}^{-}\\ \;+2.054{\text{H}}^{+}+1.022{\text{CO}}_{2}+0.0559{\text{CH}}_{4}+1.119\text{X}[\text{2H}] \end{array}$$

Equation 6: Generalization of Equation 5 for proportions of H_2_ incorporation into reductive acetogenesis between 0 and 1 (0 ≤ *y* ≤ 1)

$$\begin{array}{l} {\text{C}}_{6}{\text{H}}_{12}{\text{O}}_{6}+(0.6-0.447y){\text{H}}_{2}\text{O}+1.119X\to \\ \;(1.186+0.224y){\text{CH}}_{3}{\text{COO}}^{-}+0.475{\text{CH}}_{3}{\text{CH}}_{2}{\text{COO}}^{-}\\ \;+0.169{\text{CH}}_{3}{\text{CH}}_{2}{\text{CH}}_{2}{\text{COO}}^{-}+(1.831+0.224y){\text{H}}^{+}\\ \;+(1.469-0.447y)\;{\text{CO}}_{2}+0.895(1-y){\text{H}}_{2}+0.0559{\text{CH}}_{4}\\ \;+1.119\text{X}[2\text{H}] \end{array}$$

Equation 7: Fermentation of glucose to acetate

$${\text{C}}_{6}{\text{H}}_{12}{\text{O}}_{6}+2{\text{H}}_{2}\text{O}\to 2{\text{CH}}_{3}{\text{COO}}^{-}+2{\text{H}}^{+}+2{\text{CO}}_{2}+4\times [2\text{H}]$$

Equation 8: Acetate to propionate shift in ruminal fermentation

$${\text{CH}}_{3}{\text{COO}}^{-}+{\text{CO}}_{2}+3{\text{H}}_{2}\to {\text{CH}}_{3}{\text{CH}}_{2}{\text{COO}}^{-}+2{\text{H}}_{2}\text{O}$$

Equation 9: Incorporation of 100% H_2_ in Equation 3 into propionate production

$$\begin{array}{l} {\text{C}}_{6}{\text{H}}_{12}{\text{O}}_{6}+0.00339{\text{H}}_{2}\text{O}+1.119\text{X}\to 0.888{\text{CH}}_{3}{\text{COO}}^{-}\\ \;+0.773{\text{CH}}_{3}{\text{CH}}_{2}{\text{COO}}^{-}+0.169{\text{CH}}_{3}{\text{CH}}_{2}{\text{CH}}_{2}{\text{COO}}^{-}\\ \;+1.831{\text{H}}^{+}+1.171{\text{CO}}_{2}+0.0559{\text{CH}}_{4}+1.119\text{X}[2\text{H}] \end{array}$$

Equation 10: Generalization of Equation 9 for proportions of H_2_ incorporation into propionate production between 0 and 1 (0 ≤ *z* ≤ 1)

$$\begin{array}{l} {\text{C}}_{6}{\text{H}}_{12}{\text{O}}_{6}\text{​}+\text{​}(0.6-0.597z){\text{H}}_{2}\text{O​}+\text{​}1.119\text{X}\to (1.186-0.298z)\\ \;{\text{CH}}_{3}{\text{COO}}^{-}+(0.475+0.298z){\text{CH}}_{3}{\text{CH}}_{2}{\text{COO}}^{-}\\ \;+0.169{\text{CH}}_{3}\;{\text{CH}}_{2}{\text{CH}}_{2}{\text{COO}}^{-}+1.831{\text{H}}^{+}\\ \;+(1.469-0.298z){\text{CO}}_{2}+0.895(1-z){\text{H}}_{2}+0.0559{\text{CH}}_{4}\\ \;+1.119\text{X}[2\text{H}] \end{array}$$

Equation 11: Theoretical fermentation of glucose to acetate and propionate

$$\begin{array}{l} {\text{C}}_{6}{\text{H}}_{12}{\text{O}}_{6}\to 0.667{\text{CH}}_{3}{\text{COO}}^{-}+1.333{\text{CH}}_{3}{\text{CH}}_{2}{\text{COO}}^{-}\\ +2{\text{H}}^{+}+0.667{\text{CO}}_{2}+0.667{\text{H}}_{2}\text{O} \end{array}$$
