# Corrigendum: A theoretical comparison between two ruminal electron sinks

**DOI:** 10.3389/fmicb.2014.00235

**Published:** 2014-05-16

**Authors:** Emilio M. Ungerfeld

**Affiliations:** Visiting Scientist, Centro Regional Remehue, Instituto de Investigaciones AgropecuariasOsorno, Chile

**Keywords:** rumen, methane, hydrogen, fermentation, reductive acetogenesis, propionate, ruminant nutrition

## Conflict of interest statement

The author declares that the research was conducted in the absence of any commercial or financial relationships that could be construed as a potential conflict of interest.

